# Editorial: Recent Empirical Research and Methodologies in Defense Mechanisms: Defenses as Fundamental Contributors to Adaptation

**DOI:** 10.3389/fpsyg.2021.802602

**Published:** 2021-12-03

**Authors:** Mariagrazia Di Giuseppe, John Christopher Perry, Tracy A. Prout, Ciro Conversano

**Affiliations:** ^1^Department of Surgical, Medical and Molecular Pathology, Critical and Care Medicine, University of Pisa, Pisa, Italy; ^2^McGill University and the Institute of Community and Family Psychiatry, Jewish General Hospital, Montreal, QC, Canada; ^3^Ferkauf Graduate School of Psychology, Yeshiva University, Bronx, NY, United States

**Keywords:** defense mechanisms, adaptation, mental disorder, distress, assessment, DMRS

During the past 50 years, empirical research on defense mechanisms has come a long way in contributing to the empirical science. Beginning with Freud's initial description of defenses (Freud, 1894), there have been numerous suggested revisions to the theory of defenses. At this juncture, there is general agreement on the hierarchical organization of defense mechanisms, which, for instance lead to the inclusion of an Provisional Axis for Defensive Functioning in the 4th Edition of the Diagnostic and Statistical Manual for Mental Disorders (American Psychiatric Association, 1994). The impact of defensive functioning on physical and psychological well-being has been widely demonstrated. Less appreciated than research on defenses and mental disorders, are the growing body of studies of defenses and medical conditions (Martino et al., 2019, 2020; Conversano and Di Giuseppe, 2021). Recent studies have found that cancer patients with self-sacrificing defensive style had shorter disease-free intervals, shorter survival times, and a more unfavorable cancer staging at endpoint (Weihs et al., 2000; Di Giuseppe et al., 2018). Other studies (Zilikis and Dervenis, 2003; Vita et al., 2020; Marchini et al., 2021) have linked repressed conflicts to physical and mental disorders among those with a recurrent history of unexplained distress and depression. In line with the psychosomatic hypothesis, using neurotic defenses, which inhibit awareness of disturbing wishes, feelings, thoughts, experiences and memories, lead to impaired endocrine and immune functions and relate to the somatic manifestation of psychological distress (Bahnson and Bahnson, 1966; Vos and de Haes, 2007). Moreover, defense mechanisms, like other emotion regulation strategies, function as essential moderators of psychological responses to stressful life events (Conversano et al., 2020; Di Giuseppe et al., 2021; Merlo et al.) such as chronic physical and mental conditions (Perry, 1988; Sardella et al., 2021; Martino et al.; Martino et al.).

Defenses operate largely or partly outside of awareness, and their effects take innumerable forms. Both of these realities have created measurement challenges. Some measures (Gleser and Ihilevich, 1969; Plutchik et al., 1979; Bond et al., 1983) may have been based on the same motivational constructs but presented different organizing schemas (Horowitz et al., 1992). Other measures (Cramer, 1991; Lerner, 2005) have yielded scientifically valuable findings (Cramer, 2015), but limited themselves to a small number of defenses and lack of clear link to how individuals cope with internal and external conflicts and stressors (Rosso et al.). These methodological limitations have contributed for years to the confusion between defense mechanisms and coping strategies. As Cramer stated “*Coping mechanisms involve a conscious, purposeful effort, while defense mechanisms are processes that occur without conscious effort and without conscious awareness (i.e., they are unconscious). Also, coping strategies are carried out with the intent of managing or solving a problem situation, while defense mechanisms occur without conscious intentionality; the latter function to change an internal psychological state but may have no effect on external reality, and so may result in non-veridical perception, that is, in reality distortion*” (Cramer, 1998, p. 921). With continued advances in empirical research on the hierarchical nature of defense adaptation, two aspects of Cramer's definition appear incomplete. First, some defenses confuse internal conflict with external stress and lead the individual to maladaptive responses to the environment (e.g., counter-attacking rather than reflecting before acting). Second, it does not capture the partially conscious and more flexibly adaptive aspects of mature defensive functioning.

Defense mechanisms higher in the hierarchy (i.e., high adaptive defenses) do not follow the differential criteria described in Cramer's theory (Beresford, 2012). More than 30 years research with the Defense Mechanisms Rating Scales (DMRS; Perry, 1990) and its derivative measures (DMRS-Q; Di Giuseppe et al., 2014; Di Giuseppe and Perry, 2021; DMRS-SR-30; Di Giuseppe et al., 2020a) have demonstrated that individuals using mature defenses can: (1) be partially or fully aware of their activation (e.g., altruism, self-assertion, or self-observation); (2) intentionally use an adaptive defensive strategy to deal with internal conflict or stressful situations (e.g., anticipation or suppression); and (3) increase the probability of a gratifying resolution of the internal or external stressors without reality distortion (e.g., self-assertion or sublimation). In light of these findings, we affirm that the hierarchy of defense mechanisms (Vaillant, 1992; American Psychiatric Association, 1994; Perry, 2014; Di Giuseppe and Perry) is a comprehensive description of both more adaptive (i.e., mature defenses, overlapping in function with coping strategies) and less adaptive (i.e., immature and neurotic defenses) way of automatically responding. A clear result is that the systematic assessment of defenses which reflects this hierarchy of adaptation adds value to the diagnosis of mental disorders. We believe that it is an essential part (Perry et al., 2020; Conversano, 2021).

Research on personality disorders has found that specific defensive profiles are associated with personality traits and disorders (Maffei et al., 1995; Steiner et al., 2007; Presniak et al., 2010; Perry et al., 2013) and revealed a hierarchical organization of personality disorders based on the maturity of defensive functioning (Di Giuseppe et al., 2019). This confirms Kernberg's Personality Organization, which includes object representations (split vs. ambivalent objects) and reality testing (Kernberg, 1984). As highlighted by Kempe et al. by analyzing the defensive functioning of individuals with narcissistic personality it is possible to distinguish the defensive profiles of both grandiose and vulnerable narcissism (Kempe et al.). Similarly, recent studies investigated defenses in relation to attachment and mentalization show promising results. As demonstrated by Tanzilli et al. (2021), depressed patients with secure attachment showed higher reflective functioning and overall defensive maturity then those with insecure attachment. In line with these findings, Békés et al. found that the use of neurotic and immature defenses in the early phase of treatment predicted an increase in avoidant attachment over the course of treatment, whereas the use of immature non-depressive defenses (e.g., denial, rationalization) predicted a decrease in preoccupied attachment. Similarly, Hayden et al. reported that mentalization played an important role in the reduction of maladaptive defenses during inpatient therapy. However, their results showed that only maladaptive defenses decreased significantly in psychotherapy, while neurotic and mature did not increase significantly as expected. It is possible that some of these contradictory results stem from methodological issues, such as employing measures with inadequate reliability and validity. Furthermore, other research has demonstrated that specific defense are associated with psychiatric symptoms, interpersonal problems, externalizing behaviors, vulnerable sense of self, poor adjustment, suicidal ideation, and attempts (Dell'Osso et al., 2011; Boldrini et al., 2020).

Psychotherapy is important to help individuals improve their defensive functioning and outcome (Hoffman et al., 2016; Babl et al., 2019; Di Giuseppe et al., 2020b; Hersoug et al.). The proportion of mature and immature defenses change during psychotherapy and predict treatment response (Perry and Bond, 2012; Perry et al., 2020; Prout et al., 2021; Beresford et al.; de Roten et al.). Patient improvement in defensive maturity is likely to happen within the relationship with the therapist, who in turn activates and works with defenses in response to stress (Tanzilli and Gualco, 2020; Tanzilli et al., 2020). Therapists appear to utilize higher levels of mature defenses and lower levels of immature defenses compared to a community sample (Aafjes-van Doorn et al.). However, lower therapists' defensive maturity was associated with higher levels of vicarious trauma and professional doubt during the COVID-19 pandemic (Aafjes-van Doorn et al.). Further studies should investigate the impact of the interplay between patient and therapist defense mechanisms on outcome.

In addition to psychopathology research, interesting findings come from a number of recent studies conducted on general populations under stressful conditions. Maladaptive defensive responses were reported more often by younger people during the first wave of COVID-19 pandemic, while greater reliance on mature defenses was evident among older adults during the pandemic (Prout et al., 2020). These findings were confirmed by Beresford et al. who found that maturity of defensive functioning was associated with older age and it predicted lower depression levels in a large sample of adult cancer patients. Defense mechanisms are also associated with gender. Women use more neurotic and immature-depressive defenses to deal with internal or external stressors, while in similar conditions men tend to rely more on obsessional and immature-non depressive defenses (see Di Giuseppe and Perry, 2021, for review on the hierarchy of defenses). These findings were confirmed also in individuals diagnosed with gender dysphoria, indicating that the individual's dominant defensive functioning is related to the gender to which one chooses instead of the gender assigned at birth (Giovanardi et al.).

Advances in research on defense mechanisms widely demonstrate the impact of defense mechanisms in the onset, course, and amelioration of mental disorders. Despite the increasingly robust findings that defense mechanisms, and the hierarchy of adaptation, add scientific value to diagnosis, many practitioners have limited awareness of these scientific contributions. This special issue of Frontiers is dedicated to increase awareness of the relevance of these constructs to clinical practice. Although defense mechanisms are included in the Psychodynamic Diagnostic Manual as a key construct in diagnosis and personality structure (Lingiardi and Bornstein, 2017), we believe that greater understanding of patients' defensive functioning will be helpful to non-dynamically oriented clinicians as well. The science of our profession can be improved and the quality of our interventions can be individually tailored as a result of increased research on the role of defenses in the course of treatment. Research on defenses adds information about symptom severity, differential diagnosis, treatment compliance, recommended interventions, and expected prognosis. The inclusion of defensive functioning in diagnosis and case formulation (Perry et al., 2018, 2020) has the potential to enrich the overall clinical understanding of patients' mental functioning and how treatment can be tailored to meet their needs. In sum, close assessment of defenses provide some of the information needed to develop truly effective, personalized treatments (Zilcha-Mano, 2021).

## Author Contributions

All authors contributed in equal part to the Research Topic and the final draft of the manuscript.

## Conflict of Interest

The authors declare that the research was conducted in the absence of any commercial or financial relationships that could be construed as a potential conflict of interest.

## Publisher's Note

All claims expressed in this article are solely those of the authors and do not necessarily represent those of their affiliated organizations, or those of the publisher, the editors and the reviewers. Any product that may be evaluated in this article, or claim that may be made by its manufacturer, is not guaranteed or endorsed by the publisher.
